# Removal of a Fibro-cellular Tumor from the Recto-Vaginal Septum

**Published:** 1872-05

**Authors:** A. Reeves Jackson

**Affiliations:** Surgeon-in-Chief of the Woman’s Hospital of the State of Illinois; 785 Michigan ave.


Article V. — *Removal of a Fibro-cellular Tumor from the Recto-Vaginal Septum.* By A. Reeves Jackson, M.D., Surgeon-in-Chief of the Woman's Hospital of the State of Illinois.

Mary W., aged thirty-two years, was the mother of four children, the youngest of whom was five years old. Had never miscarried. She commenced menstruating at fifteen, and the discharge had always been regular and unattended with more than the usual amount of pain. Her general health had been uniformly good. Three years before—two years after the birth of her youngest child—she first became conscious of a difficulty in defecation, which steadily increased, until at last, unless the dejections were quite soft, she found it almost impossible to void them. The vagina had become so occluded as to render intercourse difficult or impracticable. The bladder was occasionally irritable, but not in a marked degree.

On passing a finger into the vagina it came at once in contact with a smooth, slightly-elastic, rounded body, which filled a large portion of the lower pelvis. Carrying the finger up behind the symphysis pubis I could feel the cervix uteri pressed upward and forward and lying upon the tumor, or rather, between it and the anterior vaginal wall. The growth was slightly movable upward, and not at all sensitive to pretty firm pressure.

An examination by the rectum elicited similar information, but here the finger passed with great difficulty between the tumor and the posterior wall of the bowel, showing clearly that the former was imbedded in the recto-vaginal wall.

On the rectal aspect of the tumor I could distinguish two or three depressions or grooves running in different directions as though it were irregularly lobed. I was unable to detect any fluctuation.

Feeling uncertain as to the nature of the growth, I pushed an exploring trocar into it through the rectal wall. The structure was tolerably dense, so that I was obliged to use considerable force. The instrument brought away only a few drops of blood.

Removal was decided upon, and after two days, which were passed in bed, and during which time a full dose of purgative medicine was given, I proceeded to operate, September 16, 1865, with the assistance of Drs. A. Levering and P. M. Bush. The patient declined taking an anæsthetic, thinking she would be able to undergo the operation without.

Placing her on her back upon a table the knees were drawn up and held apart. I then introduced two fingers of the left hand as far as possible into the vagina. A long-handled, probe-pointed bistoury was then passed up between the fingers, and the knife, guarded and guided by the fingers, was pressed firmly against the tumor, making, as it was withdrawn, an incision about three inches long, extending through the vaginal wall. By means of a separator, consisting of a blade of ivory somewhat resembling an overgrown finger-nail attached to a strong handle, and the fingers of first one hand and then the other, I endeavored to separate the capsule from the growth in every direction.

To a certain extent this process of enucleation was not difficult, but I found after a time that in order to complete the operation it would be necessary to introduce the entire hand into the vagina. The attempt to do this gave the patient so much pain that she at last consented to take chloroform, which was accordingly given. After she became unconscious I succeeded in dilating the vaginal orifice sufficiently to enable me to introduce the hand far enough to complete the enucleation, the last steps being greatly facilitated by passing a finger into the rectum and pressing the tumor forward and downward. In this way much aid was given also in the expulsion of the growth from the vagina which was accomplished before the patient awoke.

The tumor was about the size of an orange, and weighed seven ounces. It was composed of three lobes of varying size separated by well-defined partition walls, the situations of which were marked on the surface by distinct sulci. On cutting into the growth the basis-substance was seen to be of a yellowish-green color, and of varying degrees of density, having in some places a whitish solid appearance, and in others seeming loose and œdematous. White shining bands were seen traversing this substance in various directions, mostly irregularly, but in some parts assuming a wavy form. The cut surface, after being exposed to the air a few minutes, exhibited small drops of serous fluid oozing from the more succulent parts, and making more distinct the white bands of fibrous tissue as these latter became more consolidated.

Very little bleeding occurred during the operation, and the patient rallied satisfactorily from the effects of the chloroform. The edges of the vaginal wound were united by four silver-wire sutures—too small a number, as I afterwards thought, but the incision appeared to close so naturally that any retentive means seemed almost unnecessary.

The vagina was syringed daily with tepid water, and the bowels were confined by the exhibition of quarter grain doses of morphia every morning and evening. The diet consisted exclusively of soda crackers and green tea. The patient suffered considerably during the first four days from flatulence, but otherwise had no inconvenience except from slight pain.

22nd—9 A. M. Did not sleep well last night. Complains of more pain, which she locates in the rectum, and which she thinks would be relieved by an evacuation. Pulse quick; skin hot and dry; tongue covered with a whitish fur. Order a full dose of castor oil, to be followed in four hours by an enema of diluted ox-gall. Also to have cooling medicines during the day.

7 P. M. Find her much relieved. On the introduction of the syringe pipe she had felt a sudden sharp pain, which was immediately followed by a discharge of two or three teaspoonfuls of pus. It was plain enough now. The nurse had opened an abscess—exactly what I ought to have done ten hours before. The bowels had been freely moved, and the febrile symptoms had nearly disappeared.

A few days afterwards the patient informed me that intestinal gas was escaping from the vagina, and that on one occasion a small quantity of feculent matter had also taken this course. An examination revealed a recto-vaginal fistula at the site of the abscess, the opening being sufficiently large to permit the passage of the little finger.

I made an application of nitrate of silver to the edges of the fistula on three occasions during the time the patient remained under my care, with the effect of lessening somewhat the size of the opening.

She returned to her home (nearly sixty miles distant) and I did not see her again until the following July, when she returned, by advice of her physician, for the purpose of having an operation done for the cure of the fistula. Nitrate of silver and other stimulating applications had been sedulously used, at intervals of a few days, but without success.

The usual operation was performed. The patient was placed on her back, and the parts being in a good light, the fistula was exposed by means of a bivalve speculum placed in the vagina.

The edges of the opening were freely pared, and beveled at the expense of the vaginal mucous membrane. They were then brought together and retained by means of six silver sutures. The subsequent treatment consisted in keeping the bowels quiet by the daily administration of opium and the cleansing of the vagina by irrigations of tepid water. The sutures were removed on the tenth day, and union was found complete.

785 Michigan ave.

